# The Stranded Stone: Relationship Between Acute Appendicitis and Appendicolith

**DOI:** 10.4103/1319-3767.56106

**Published:** 2009-10

**Authors:** Ahmad Aljefri, Nizar Al-Nakshabandi

**Affiliations:** Department of Radiology, King Fahad Medical City, Saudi Arabia; 1 Department of Radiology, King Khalid University Hospital, King Saud University, Saudi Arabia

**Keywords:** Appendicolith, appendicitis, CT scan

## Abstract

**Background/Aim:**

To examine the relationship between acute appendicitis and the presence of an appendicolith in abdominal CT scans of patients attending emergency services.

**Materials and Methods:**

Abdominal CT scan reports were retrospectively reviewed for 267 patients through the PACS database. A 16-slices MDCT GE Light Speed scanner (Milwaukee WI) was used with a scanning protocol of 5 mm axial collimation and a pitch of 1.0, along with oral contrast material (Gastrografin 3.7% diatrizoate meglumine) and 140 mL of intravenous (IV) nonionic contrast material (Omnipaque). Particular attention was given to the study protocol, patients' age, and gender.

**Statistical Analysis:**

We used MS-EXCEL and SPSS version 12.0 to perform chi-square and Fisher's exact tests. Bookends and Papers, components in Mac OS X software, were used for literature reviews and the organization of results.

**Results:**

Two hundred and sixty-seven abdominal CT scan reports were examined along side their respective images on a GE Centricity workstation. Thirty-four (12.7%) were labeled as acute appendicitis cases based on the CT findings and the rest were assigned other diagnoses. Twenty-six of the 267 CT scan reports were plain studies and 241 were contrast-enhanced scans. Less than half of the patients (123, 46.1%) were males and 144 (53.9%) were females. Thirteen males (48.1%) and 14 (51.9%) females were found to have an appendicolith. Only 3% in the ≤ 11 years' age group, in contrast to 40% in the 11-20 years' age group, was diagnosed with appendicitis. The incidence in other age groups was as follows: 19% in the 21-30, 14% in the 31-40, 2.5% in the 41-50, 8% each in the 51-60 and 61-70, and none in the ≥71 years' age groups.

**Conclusions:**

We conclude that the presence of an appendicolith i) has no particular predilection for gender or age, and ii) is not associated with a diagnosis of appendicitis.

Acute abdominal pain imposes a diagnostic and therapeutic dilemma in emergency departments across the globe. There is a myriad of pathologies that can present with acute abdominal pain, the most frequent one requiring surgical attention being acute appendicitis. Up to one third of acute appendicitis cases have atypical presentation, especially in the pediatric population.[1,2] Among the current advances in diagnostic imaging, computed tomography (CT) scan is gaining popularity in the diagnosis of acute appendicitis. The rate of false positive cases has decreased from 20% with negative appendectomy to 7% with the introduction of CT imaging into clinical practice.[3] Many clinical and imaging criteria have been described for the diagnosis of acute appendicitis. Using the presence of an appendicolith as a sole criterion for the diagnosis of acute appendicitis is still considered to be controversial. In this retrospective study, we examined the relationship between acute appendicitis and the presence of an appendicolith in abdominal CT scans of patients attending emergency services in a tertiary care center in Riyadh, Saudi Arabia. We also tried to understand the relationship between the presence of an appendicolith and the incidence of appendicitis with respect to patients' age and gender.

## MATERIALS AND METHODS

A total of 267 abdominal CT scan reports were retrospectively reviewed for patients attending the King Fahd Medical City emergency service between May 2007 and May 2008, through the picture archival and retrieval system (PACS) database. A 16-slices MDCT GE Light Speed scanner (Milwaukee WI) was used with a scanning protocol of 5 mm axial collimation and pitch of 1.0 for the nonenhanced scans. The enhanced scans were done in a manner similar to that of the nonenhanced scans, except that 1000 mL of an oral contrast material (Gastrografin 3.7% diatrizoate meglumine) was given along with the injection of 140 mL of an IV nonionic contrast material (Omnipaque). Furthermore, each examination was reviewed using a bone window to enhance the detection of an appendicolith.[4–6] Particular attention was given to the study protocol, and patients' age and gender. Several keywords were scanned in each report, including appendiceal enhancement, wall thickening, periappendiceal fat stranding, free peritoneal and pelvic fluid, and the presence of an appendicolith. We used MS-EXCEL and SPSS version 12.0 to perform chi-square and Fisher's exact tests. The Chi square test was used to analyze relationships between the presence of an appendicolith and the incidence of appendicitis whereas Fisher's exact test was used to analyze the relationship between the presence of appendicolith and patients' age as well as gender. Bookends and Papers on Mac OS X software were used for literature review and the organization of results.

## RESULTS

**Figure 1:**
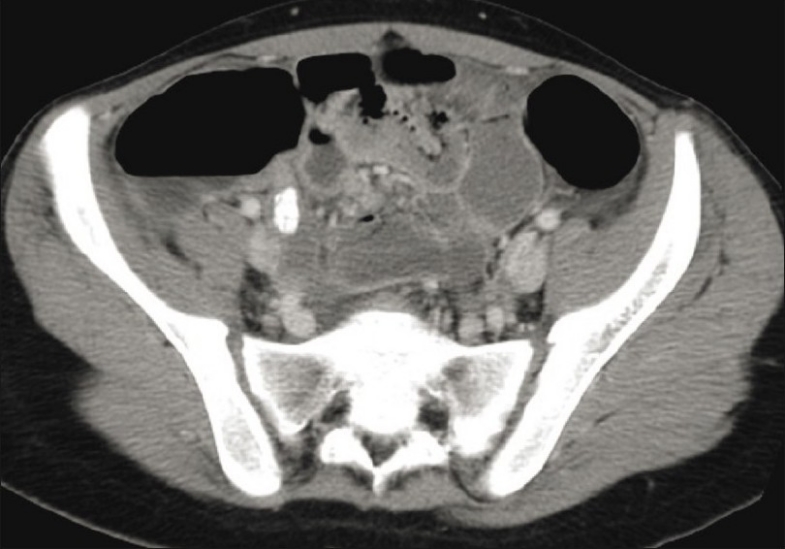
An 18 year-old female with right lower quadrant pain. CT scan with oral and IV contrast shows an appendicolith in an inflamed appendix

Two hundred and sixty-seven abdominal CT scan reports were examined alongside their respective images on a GE Centricity PACS (Picture Archival and Retrieval System) workstation. Of these scans, 34 (12.7%) were labeled as acute appendicitis cases based on the CT findings. Thirteen males (48%) and 14 females (51.9%) were found to have an appendicolith [Figure 1], while the remaining 7 patients could not be obtained. The rest of the patients were given other diagnoses. Appendicoliths were found in two plain scans (7.7%) compared to 32 contrast-enhanced scans (13.28%). Out of the 267 cases examined, 123 were males (46%) and 144 were females (54%). Only 3% in the ≤11 years' age group, in contrast to 40% in the 11-20 years' age group, were diagnosed with appendicitis. The incidence in other age groups was as follows: 19% in the 21-30, 14% in the 31-40, 2.5% in the 41-50, 8% each in the 51-60 and 61-70, and none in the ≥71 years' age groups.

## DISCUSSION

The appendicolith, also known as “fecolith” or “corpolith”, represents calcified deposits in the appendix, and contributes to the pathogenesis of acute appendicitis. It is defined as an area of high attenuation measuring ≤ 1 cm that is located in the pericecal areas, or in cases of perforation in the Morrison's (Douglas) pouch.[4] Case reports of the prescence of an appendicolith and its strong correlation to acute appendicitis can be found in literature.[5,6] The appendicolith has been detected by using various modalities ranging from plain abdominal radiography and ultrasound examination to computed tomography.

Although the appendicolith has a significant role to play in the pathogenesis of acute appendicitis, it is not the sole entity in its pathogenesis. Other causes of luminal obstruction have been described: Lymphoid hyperplasia, foreign bodies, strictures, tumors, and Crohn's disease.[7] Although the pathogenesis of the formation of an appendicolith is still unknown, several case reports have mentioned sources such as an ingested foreign body or a dislodged gall stone eroding through the gall bladder.[8] Appendicoliths represent homogeneous or laminated calcification in up to 25% of all cases.

The presence of an appendicolith per se is not considered diagnostic for acute appendicitis in the absence of pericecal inflammatory changes or appendiceal wall enhancement. Of all the CT signs of acute appendicitis, the presence of appendicolith(s) has been reported to have 100% specificity but low sensitivity (44%).[9] It has been reported in literature that 28% of adult and 30% of pediatric patients with acute appendicitis have appendicoliths.

CT findings of abscess, extraluminal gas, and ileus have the highest specificity but low sensitivity[10] in comparison to the detection of an intraluminal appendicolith which has low sensitivity and specificity in the detection of perforation. Moreover, Huwart and El-Khuory *et al*. have studied abdominal CT scans of 85 adult subjects without any known symptoms related to the gastrointestinal tract. They found that 57/85 patients had not undergone appendectomy, but an appendicolith was detected in 13% of all these subjects. Hence; they concluded that there was no statistical significance of the presence of appendicolith in the diagnosis of acute appendicitis.[11] In contrast, Jabra *et al*. who had studied the diagnosis of appendicitis in children using CT scans, reported that appendicoliths could be an incidental finding on an abdominal radiograph done for other purposes. However, when associated with abdominal pain, there is 90% probability of acute appendicitis in patients[12] besides a 50% higher risk of appendiceal perforation. Several authors have described diagnostic criteria based on the imaging modality for acute appendicitis, however, they did not include appendicoliths among these criteria.

Other than its diagnostic significance, the presence of an appendicolith has significant therapeutic considerations. The treating surgeon must be forewarned for it if the patient has any surgical interventions. In several studies as well as case reports, dropped appendicoliths have been described to contribute to the overall morbidity of patients. Pelvic abscesses have been reported from dropped appendicoliths, especially with laparoscopy appendectomy. Retrieval options include an open surgical approach, laparoscopic retrieval, and CT-guided retrieval.[1,10,13–18]

Although controversial, the finding of an appendicolith may be sufficient evidence to perform a prophylactic appendectomy in asymptomatic patients, given the higher rate of perforation at the time of acute appendicitis.

In this study, we did not group patients according to age due to techniqual difficulty. In addition, the final pathological diagnosis was not obtained due to limited resources.

Although the presence of an appendicolith in the absence of other findings such as thickened appendix or periappendiceal infiltration, is not diagnostic for appendicitis, it could be related to prior appendicitis. Old healed appendicitis must be distinguished from chronic appendicitis; the latter could benefit from curative surgery.

## CONCLUSION

From the data obtained and the reviewed literature, we conclude that the presence of an appendicolith has no particular predilection to gender or age. Its correlation with the diagnosis of acute appendicitis per se was poor and should be avoided unless adjunct CT signs of acute appendicitis are demonstrated.
